# Formulation development of a biphasic release tablet-in-tablet system containing ketorolac tromethamine

**DOI:** 10.1371/journal.pone.0354689

**Published:** 2026-07-29

**Authors:** Ping Zou, Yuan Zeng, Xiang-ru Liao, Xiang-yang Xie, Yin-ke Li, Yi-hui Ma, Hui Liu

**Affiliations:** 1 Department of Pharmacy, The Central Hospital of Wuhan, Tongji Medical College, Huazhong University of Science and Technology, Wuhan, China; 2 Key Laboratory for Molecular Diagnosis of Hubei Province, The Central Hospital of Wuhan, Tongji Medical College, Huazhong University of Science and Technology, Wuhan, Hubei, China; 3 Department of Pharmacy, General Hospital of Central Theater Command, Wuhan, China; 4 College of Nursing and Health Management & College of Life Science and Chemistry, Wuhan Donghu University, Wuhan, China; 5 Department of Stomatology, General Hospital of Central Theater Command, Wuhan, China; 6 Department of Clinical Pharmacy, General Hospital of Central Theater Command, Wuhan, China; National University of Rosario, ARGENTINA

## Abstract

Ketorolac tromethamine (KT) is a potent nonsteroidal anti-inflammatory drug (NSAID) used for moderate to severe pain management. Its short elimination half-life necessitates frequent dosing, which can reduce patient compliance. The objective of this study was to formulate, develop, and optimize a novel biphasic-release tablet-in-tablet (TIT) system for KT to provide immediate pain relief followed by extended drug release. A TIT system was designed, comprising an immediate-release (IR) outer layer and an extended-release (ER) core matrix. The ER core was optimized using a 3^2^ full factorial design, with the amounts of HPMC K100M and HPMC K4M as independent variables. Formulations were evaluated for pre- and post-compression parameters, drug-excipient compatibility, and *in vitro* drug release. The drug release kinetics and mechanism were analyzed using various mathematical models. The optimal ER core formulation (F1) exhibited a drug release of 4.6% at 0.5 h and 66.1% at 12 h, closely matching the theoretical profile (similarity factor *f*_2_ = 51). Release kinetics followed the first-order model and the Korsmeyer-Peppas model indicated an anomalous (non-Fickian) release mechanism (n = 0.831). The corresponding TIT formulation (F11) demonstrated desired physicochemical properties: hardness of 75 N, friability of 0.59%, and rapid outer layer disintegration (<50 s). The TIT provided an initial burst release (~30%) within 30 minutes, followed by sustained release exceeding 90% over 24 hours. This TIT system represents a promising alternative to conventional KT tablets for effective pain management.

## 1. Introduction

Ketorolac tromethamine (KT) is a widely used nonsteroidal anti-inflammatory drug (NSAID) known for its efficacy in managing moderate to severe pain, particularly in postoperative settings. As a potent analgesic [1], KT primarily exerts its therapeutic effects by inhibiting the cyclooxygenase enzymes COX-1 and COX-2, thereby providing significant pain relief comparable to opioids and corticosteroids. It is extensively utilized across various clinical stages, including preoperative, intraoperative, and immediate postoperative phases, for pain relief in abdominal, gynecologic, oral, orthopedic, and urologic surgeries. KT is also indicated for the management of acute renal colic pain, as well as visceral pain related to cancer [2].

The use of non-opioid analgesics, such as KT, in perioperative pain management is increasingly encouraged to reduce or eliminate opioid consumption in postoperative care, addressing the growing concerns regarding opioid dependence. KT is classified as a Class I drug in the Biopharmaceutical Classification System (BCS) [3], indicating good aqueous solubility and *in vivo* permeability. Despite its favorable absorption properties, KT has a relatively short plasma elimination half-life of approximately 4–6 hours [4]. It is commercially available in several formulations, including injectable solutions (15−30 mg), oral tablets (10 mg), and ophthalmic solutions (0.4–0.5%) [1].

Due to its short half-life, KT requires frequent dosing to maintain therapeutic plasma concentrations and ensure consistent analgesic effects. In clinical practice, it is typically administered in oral doses of 10–20 mg, twice daily, to sustain adequate therapeutic levels. However, the frequent dosing required for effective pain control, along with the resultant fluctuations in plasma drug concentrations, can limit the clinical utility of existing dosage forms. Recent studies have highlighted the potential of continuous intravenous infusion of ketorolac as an effective option for postoperative analgesia, particularly in older patients [5], offering an alternative to traditional dosing regimens. Research involving 12 healthy male subjects compared two treatment regimens—continuous subcutaneous infusion and repeated intramuscular injections—showing similar pharmacokinetic profiles for both, though the peak plasma concentration was lower with continuous infusion, and both treatments were well-tolerated [6]. Previous research on the risk of gastrointestinal bleeding linked to NSAIDs in both animals [7] and humans [8] has shown that sustained-release formulations of bermoprofen and flurbiprofen are not only effective but also well-tolerated, with a reduced incidence of gastrointestinal side effects.

Effective management of postoperative pain is critical for promoting rapid recovery and improving overall patient outcomes. Proper pain control not only alleviates discomfort but also accelerates healing, restores mobility, and minimizes the risk of complications following surgery. Postoperative pain management is typically tailored to individual patient needs, with an initial emphasis on multimodal, non-opioid analgesics. Although opioid analgesics are commonly used for acute postoperative pain relief [9,10], their long-term use is associated with significant drawbacks, including the development of tolerance, physical and psychological dependence, withdrawal symptoms, and other adverse effects [11]. Consequently, NSAIDs, such as KT, are frequently preferred for managing postoperative pain due to the absence of these opioid-related complications.

**Fig 1:**
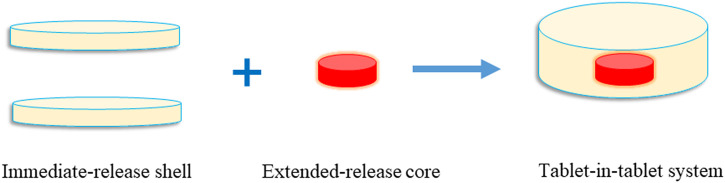
Schematic diagram of the tablet-in-tablet (TIT) system of ketorolac tromethamine.

To optimize the dosing regimen of KT and improve its therapeutic efficacy, a novel extended-release (ER) formulation has been developed. This formulation is designed to sustain drug release over 24 hours, thereby reducing dosing frequency. The system employs a tablet-in-tablet (TIT) design comprising an immediate-release (IR) outer layer and an ER core (Fig 1). The IR outer layer is intended to rapidly elevate the plasma concentration to the therapeutic level upon administration, while the ER core, acting as a sustained-release matrix tablet, ensures maintenance of therapeutic plasma concentrations for over 24 hours following the initial rapid release from the outer layer.

Compared with conventional bilayer tablets, in which two layers are compressed together in a vertically stacked configuration, the TIT system completely encloses the core within an outer shell. This structural design provides several advantages [12]: (i) greater flexibility in adjusting the dose ratio between the core and the outer layer; (ii) the outer layer can function as a protective barrier against environmental factors such as moisture and light; and (iii) the core may be formulated as a controlled-release matrix without interference from the outer layer during drug release. Although bilayer tablets are generally simpler to manufacture, the TIT system ensures superior core isolation and may provide improved mechanical integrity.

This innovative TIT formulation aims to streamline the dosing regimen while improving both tolerability and efficacy. By achieving a more consistent plasma concentration profile, the formulation reduces the occurrence of super- or subtherapeutic plasma levels commonly observed with multiple daily doses of IR formulations [13]. Through precise control over the release kinetics, this TIT formulation has the potential to optimize treatment outcomes, minimize adverse effects, and ultimately enhance the overall therapeutic experience for patients. In contrast to a previously reported KT TIT formulation that employs an osmotic pump tablet for extended release [14,15], our design here will utilize an ER matrix tablet as the core of the TIT system offers a simpler and more efficient approach.

In this study, we employed the TIT technique to develop a novel KT ER formulation, which comprises an ER core tablet and an IR outer shell, both compressed into a single-unit dosage form. The core tablet contains KT as the active pharmaceutical ingredient, microcrystalline cellulose (MCC) as a filler, and hydroxypropyl methylcellulose (HPMC) as part of the drug matrix. The controlled release properties of the formulation are modulated by adjusting the ratio of MCC to HPMC. The IR outer shell, which includes KT, MCC, and cross-linked sodium carboxymethyl cellulose (CMC) as a disintegrant, is designed for rapid drug release within 30 min. This outer shell provides an initial dose to quickly achieve therapeutic drug concentrations, while the ER core ensures the gradual release of the drug over an extended period. The dissolution profiles of KT from these formulations were evaluated *in vitro*, and the release data were analyzed using various mathematical models to understand the release kinetics and optimize the formulation for future therapeutic efficacy.

## 2. Materials & methods

### 2.1. Materials

Ketorolac tromethamine (KT) with a purity exceeding 99%, was provided by Hainan Zhuoke Pharmaceutical Co. (China). Hydroxypropyl methylcellulose (HPMC) was sourced from Shanghai Colorcon Coating Technology Co., Ltd. (China). Microcrystalline cellulose (MCC) and cross-linked sodium carboxymethyl cellulose (CMC) were obtained from Asahi Kasei Chemicals Corporation (Japan). Both magnesium stearate (Mg stearate) and silicon dioxide (SiO_2_) were supplied by Anhui Sunhere Pharmaceutical Excipients Co., Ltd. (Anhui, China). All other chemicals and materials used in this study were acquired from Sinopharm Chemical Reagent Co., Ltd. (Shanghai, China), meeting analytical or HPLC grade standards.

### 2.2. Calculation of tablet dose

The drug release rate from TIT is assumed to be independent of the remaining drug quantity in the tablet and to follow a constant rate over time, consistent with zero-order release kinetics. The release profile of the TIT is described by zero-order kinetics, as illustrated in Eq. (1) [16].


$$\begin{array}{c} {\text{K}}_{\text{0r}}=\text{Rate in}=\text{Rate out}={\text{K}}_{\text{e}}\times \text{Cd }\times {\text{V}}_{\text{d}} \end{array}$$ (1)

where, “Rate in” represents the rate at which the drug becomes available in the body, “Rate out” refers to the rate at which the drug is eliminated from the body, K_0r_ is the zero-order rate constant for drug release (amount/time), K_e_ is the first-order rate constant of overall drug elimination (h^−1^), C_d_ is the desired plasma concentration of the drug at steady state, and V_d_ is the volume of distribution.

The rationale for selecting the desired steady-state plasma concentration (C_d_ = 0.59 mg/L) is based on published clinical pharmacokinetic data for KT. At this concentration, KT achieves effective analgesic activity with a favorable safety profile, as established in clinical studies [4]. The release duration of 24 hours was selected to achieve once-daily dosing, which would significantly improve patient adherence compared to the conventional 4–6 times daily dosing schedule. The volume of distribution (V_d_ = 12.25 L) and elimination half-life (t_1/2_ = 5.3 h, K_e_ = 0.69/t_1/2_ = 0.1302 h^−1^) are well-established pharmacokinetic parameters for KT [4,17]. Therefore, the drug release rate (K_0r_) is calculated to be 0.9410 mg/h (Eq. (1)) [17]. To maintain a steady-state condition in the bloodstream, the rate constant for drug release (K_0r_) must be equal to the elimination rate constant (K_e_). Given that the drug is completely absorbed from the gastrointestinal tract, with an oral bioavailability of 100%, the drug release rate is set to match the elimination rate, which is 0.9410 mg/h.

For a system where the maintenance dose releases the drug at a zero-order rate over a specified period, the total dose required can be calculated using Eq. (2) [14].


$$\begin{array}{c} \text{W}=[{\text{D}}_{\text{i}}-({\text{K}}_{\text{0r}}\times {\text{T}}_{\text{p}})]\;+{\text{K}}_{\text{0r}}\times {\text{T}}_{\text{d}} \end{array}$$ (2)

where, W is the total dose, D_i_ is the initial dose, K_0r_ is the zero-order rate constant, T_p_ is the time to reach peak plasma drug concentration, and T_d_ is the total duration (in hours) for which the drug is to be released from the TIT.

The initial dose (Dᵢ) was set at 10 mg, which is the standard oral dose of KT IR tablets [17]. Based on Eq. (2), with Tₚ = 0.733 h, T_d_ = 24 h, and K₀ᵣ = 0.9410 mg/h, the total dose was calculated as 31.89 mg, approximately 32 mg. Therefore, the IR dose was 10 mg and the ER dose was 22 mg (rounded to 21.89 mg for formulation convenience).

### 2.3. Calculation of theoretical release profile

For the 24-hour biphasic release formulation of KT, the drug is expected to release 10 mg during the first half an hour (<44 min), similar to a conventional tablet, which corresponds to 31.2% (w/w) of the total dose (32 mg). For the remaining 23.5 hours, the drug should be released at a rate of 0.94 mg per hour, equating to 2.9% (w/w) of the total dose per hour.

### 2.4. Fourier transform infrared spectroscopy (FTIR)

Infrared analysis was conducted using a WQF-530A FTIR spectrometer (Beijing Beifen-Ruili Analytical Instrument Co., Ltd., China), with potassium bromide as the background. The samples were scanned over a range of 4000–400 cm⁻^1^ at a scan speed of 1 cm/s. Spectra were recorded at room temperature for KT, excipients, and their physical mixtures.

### 2.5. Differential scanning calorimetry (DSC)

DSC curves for KT, excipients, and their physical mixtures were obtained using an HSC-1 differential scanning calorimeter (Beijing Hengjiu Experimental Equipment Co., Ltd., China). The purge gas used was dry nitrogen, flowing at a rate of 80 mL/min and a pressure of 0.2 MPa. Approximately 4 mg of each sample was heated at a rate of 10°C/min within the temperature range of 25°C to 250°C. The DSC curves were generated using a closed aluminum crucible, with an empty aluminum crucible serving as the control.

### 2.6. Powder X-ray diffraction (PXRD)

The crystalline structure of KT, excipients, and their physical mixtures was analyzed using a D8 Advance X-ray powder diffractometer (Bruker, Germany). Diffraction patterns were recorded over a 2θ range of 10° to 80°, using a copper X-ray source (K_α_ = 0.15406 nm) and a nickel β filter, at a current of 40 mA and a voltage of 40 kV. The instrument was operated in step-scan mode with a step size of 0.02° and an integration time of 0.5 s per step.

### 2.7. Drug-excipient compatibility

To comprehensively evaluate the compatibility of KT with excipients, three complementary techniques were employed: FTIR (to detect chemical interactions), DSC (to assess thermal behavior and possible solid-state changes), and PXRD (to monitor crystalline form alterations). Together, these methods provide a complete picture of both chemical and physical compatibility, ensuring that the drug remains stable and unchanged in the formulation. The study involved the preparation of physical mixtures, each containing KT and five specific excipients in a 1:1 (W/W) ratio [18].

The tested physical mixtures were: KT + HPMC + MCC + CMC + SiO_2_ + Mg stearate.

These mixtures were analyzed before being subjected to controlled storage conditions. All samples were stored for 15 days under controlled conditions (40°C ± 2°C, relative humidity (RH): 75% ± 5%). Throughout this period, visual inspections were performed to identify any signs of clumping, liquefaction, color changes, or the presence of unusual odors or gas emissions. After the storage phase, a second round of spectral analyses was conducted. The FTIR, DSC, and PXRD spectra of pure KT were compared with those of the physical mixtures to evaluate potential interactions between the drug and the excipients.

### 2.8. Tablet preparation

A multilayer tablet press is used to compress KT TIT formulation consisting of ER core tablet surrounded by IR outer layers. In the preparation process, each component was accurately weighed and blended using a geometric dilution method for the ER core tablet. The resulting mixtures were then passed through a fine mesh sieve with an 80-mesh size, followed by thorough mixing in a Turbula T2F blender (Willy A. Bachofen AG, Switzerland) for a duration of 20 min. Afterward, the mixtures were dried at 40°C for 4 h. The dried powders of ER core tablet were then compressed into core tablets using an 8 mm (diameter) round concave punch on a KTP 1X tablet press (Romaco Holding GmbH, Germany) under a punch pressure of 3.5 kN. Once compressed, the core tablets were filled into a tablet cartridge. Nine distinct formulations were prepared, as outlined in Table 1.

**Table 1:** Formulation for extended-release core tablets of ketorolac tromethamine.

Ingredients (mg)	Formulation Codes								
	F1	F2	F3	F4	F5	F6	F7	F8	F9
KT	22.6	22.6	22.6	22.6	22.6	22.6	22.6	22.6	22.6
HPMC K100M	30	30	30	20	20	20	10	10	10
HPMC K4M	30	20	10	30	20	10	30	20	10
MCC PH101	36.4	46.4	56.4	46.4	56.4	66.4	56.4	66.4	76.4
SiO_2_	0.5	0.5	0.5	0.5	0.5	0.5	0.5	0.5	0.5
Mg stearate	0.5	0.5	0.5	0.5	0.5	0.5	0.5	0.5	0.5
*Total Weight*	120	120	120	120	120	120	120	120	120

KT: ketorolac tromethamine, HPMC: hydroxypropyl methylcellulose, MCC: microcrystalline cellulose, Mg stearate: magnesium stearate, SiO_2_: silicon dioxide.

Each batch was prepared to yield 100 tablets.

The powder mixture used to coat the core tablets (Table 2) were formulated for rapid drug release. To facilitate this, half of the outer layer (IR layer) powders were placed in a mold and pre-compressed using a 12 mm (diameter) round concave punch at 2.0 kN. This formed the bottom layer, which served as the foundation for the core tablet. The ER core tablet was then automatically positioned into the mold, and the remaining rapid-release powders were added before being compressed again using the same punch at 11.0 kN. The compression force was precisely controlled to ensure that the final tablets exhibited a hardness within the range of 50–85 N.

**Table 2:** Formulation of ketorolac tromethamine tablet-in-tablet.

Type of layer	Ingredients (mg)	F10	F11	F12
Core tablet	KT	22.6	22.6	22.6
	HPMC K100M	30	30	30
	HPMC K4M	30	30	30
	MCC PH101	36.4	36.4	36.4
	SiO_2_	0.5	0.5	0.5
	Mg stearate	0.5	0.5	0.5
Outer layer	KT	9.4	9.4	9.4
	MCC	280	310	340
	CMC	9.6	10.6	11.6
	SiO_2_	1	1	1

KT: ketorolac tromethamine, HPMC: hydroxypropyl methylcellulose, MCC: microcrystalline cellulose, PEO: polyethylene oxide, SiO_2_: silicon dioxide, CMC: cross-linked sodium carboxymethylcellulose. The outer layer weights listed are for the shell only (excluding the core). Total tablet weight = core weight (120 mg) + outer layer weight. For example, F11 has an outer layer of 331 mg, giving a total weight of 451 mg (as shown in Table 10).

Each batch was prepared to yield 100 tablets.

### 2.9. Experimental design

To assess and use the effect of polymer ratios on the drug release performance of ER core-tablets, a 3^2^ Full Factorial Design with Response Surface Methodology (RSM) was employed for optimizing the ER core tablet formulation. In this experiment, the amounts of HPMC K100M (X_1_) and HPMC K4M (X_2_) were chosen as independent variables. These independent variables were classified into three levels: low (−1), medium (0), and high (+1), leading to the creation of 9 distinct formulations (Tables 3 and 4). The *in vitro* drug release characteristics were assessed by three dependent variables: Y_1_ (drug release at 0.5 h) and Y_2_ (drug release at 12 h).

**Table 3:** Independent variables and their coded and actual levels in the 3² full factorial design.

Independent variable	Level (−1)	Level (0)	Level (1)
X_1_: HPMC K100M (mg)	10	20	30
X_2_: HPMC K4M (mg)	10	20	30

**Table 4:** Layout of the three-level factorial response surface design and observed responses for the core-tablets.

Run	Batch codes	Coded values		Actual values		Responses	
		X_1_	X_2_	X_1_	X_2_	Y_1_	Y_2_
4	F1	1	1	30	30	4.6	66.1
9	F2	1	0	30	20	8.3	74.6
7	F3	1	−1	30	10	17.2	96.1
5	F4	0	1	20	30	12.9	82.1
6	F5	0	0	20	20	19.4	95.7
10	F6	0	−1	20	10	21.6	94.8
11	F7	−1	1	10	30	17.6	96.5
1	F8	−1	0	10	20	23.5	95.2
2	F9	−1	−1	10	10	32.7	95.4

The coded values (–1, 0, + 1) correspond to actual polymer amounts of 10, 20, and 30 mg for both HPMC K100M and HPMC K4M (see Table 2). During preparation, these actual amounts were weighed directly. The Design-Expert 12.0.3.0 software (Stat-Ease, Inc.) was used to generate the experimental matrix, analyze responses, and predict optimal factor settings based on the actual values. The statistical analysis was conducted via analysis of variance (ANOVA). A p-value of less than 0.05 was considered statistically significant.

To obtain the optimal formulation conditions, two optimization criteria were established and inputted into the Design-Expert software: (i) Y_1_ (drug release at 0.5 h) was set to ‘minimize’ to ensure the ER core did not release excessive drug during the early phase, as the IR outer layer was designed to provide the initial dose; (ii) Y_2_ (drug release at 12 h) was set to be “in range of 50-75%” to achieve a balanced release profile. The 50–75% range at 12 h was selected because it ensures adequate drug release by the midpoint of the release duration while allowing for the typical slowdown of drug release in the latter phase of hydrophilic matrix systems, thereby facilitating near-complete release by 24 h.

### 2.10. Powder evaluation

In order to evaluate the flowability of the formulation powders, a comprehensive investigation of several essential parameters was performed. This included the bulk and tapped density, Carr’s compressibility index, Hausner ratio, and the angle of repose [19]. These parameters were measured using a BT-1001 intelligent powder characterization tester (Dandong Baite Instrument Co., Ltd., China), following the guidelines outlined in the 2020 edition of the Chinese Pharmacopeia (CP).

### 2.11. Tablet evaluation

To assess the fundamental physical characteristics of the prepared ER core tablets and the final TITs, several critical parameters were measured, including tablet thickness, hardness, tablet weight, friability, and disintegration time (for the outer shell layer). These evaluations were performed according to the standards outlined in CP (2020 edition) for tablet dosage forms.

Tablet thickness was measured using a SL01–3 vernier caliper (Biaokang Instrument Co., Ltd., China). Tablet hardness was determined with a YPD 200C tablet hardness tester (Shanghai Huanghai Pharmaceutical Instrument Co., China). Tablet weight was assessed using a BT25S electronic balance (Sartorius Lab Instruments GmbH & Co. KG, Germany). Friability was measured with a FT2000-AF tablet friability tester (Tianda Tianfa Technology Co., Ltd., China). The disintegration time of the outer layer of the tablet-in-tablet system was determined using a ZB-1E disintegration tester (Tianda Tianfa Technology Co., Ltd., China).

### 2.12. Drug content determination

Twenty tablets (core tablets or outer layer) were randomly selected from each formulation batch, and their individual weights were recorded. These tablets were then triturated using a ceramic mortar. The average tablet weight was subsequently calculated. For sample preparation, the mobile phase was employed as the diluent solvent. A precisely weighed portion of the powdered tablets, equivalent to 30 mg of KT, was transferred to a 100 mL volumetric flask containing 50 mL of mobile phase. The mixture was sonicated for 30 min to ensure complete dissolution and then shaken for 24 h at room temperature. The resulting solution was subsequently diluted to a final volume of 100 mL with additional mobile phase. To achieve the desired concentration for analysis, 10 mL of the filtered solution was withdrawn and further diluted to 50 mL with mobile phase, then filtered through a 0.22-μm cellulose syringe filter. Finally, the prepared sample was injected into the high-performance liquid chromatography (HPLC) system for quantification of the drug concentration.

The concentration of KT was determined using a Shimadzu LC-20AT HPLC system (Shimadzu Corporation, Japan). The mobile phase consisted of methanol, water, and glacial acetic acid in a volumetric ratio of 65:34:1. A 20 μL sample was injected into the system via an auto-injection mechanism. Chromatographic separation was achieved on a DIKMA C_18_ column (4.6 × 250 mm, 5 μm; Dikma Technologies Inc., China), with the column maintained at 40°C. KT was detected at a wavelength of 322 nm, and the mobile phase flow rate was set at 0.9 mL/min.

The HPLC method was validated for specificity, accuracy, and both intra- and inter-day precision, in accordance with the guidelines outlined in the CP (2020 edition). All measurements were performed in triplicate, and the standard deviations were calculated to assess the precision of the results.

### 2.13. In vitro dissolution

*In vitro* drug release studies were performed using an RCZ-8 dissolution tester (Shanghai Huanghai Pharmaceutical Instrument Co., China). The dissolution profiles of KT from TITs were assessed according to the USP paddle method (Type II). The dissolution medium (900 mL) was maintained at 37°C ± 0.5°C, and the paddle speed was set at 50 rpm. During the first 2 h, the tablets were immersed into 500 ml of simulated gastric fluid (SGF; pH 1.2), followed by transfer to 400 ml of simulated intestinal fluid (SIF; pH 6.8) for the remaining duration of the experiment [20]. At predetermined time points (0.5, 1, 2, 4, 6, 8, 10, 12, 14, 16, 18, and 24 h), 2 mL samples were withdrawn from the dissolution vessels. After each sampling, an equivalent volume of fresh dissolution medium was added to replace the withdrawn volume. The drug concentrations in the sample solutions were quantified using HPLC as described above.

### 2.14. Drug release kinetics

The drug release kinetics from the optimal tablets were evaluated by fitting the *in vitro* release data to several mathematical models, including zero-order, first-order, Higuchi, and Korsmeyer-Peppas models [21]. The optimal model for describing the drug release profile was determined through the analysis of a statistical parameter with the highest coefficient of determination (R²).

### 2.15. Statistical analysis

The *in vitro* drug release profiles of the ER core tablet formulations (test) were compared with the theoretical release profile (reference) of KT ER core tablets using the similarity factor (*f*_2_) [22]. The similarity of the dissolution profiles was assessed by calculating *f*_2_, as defined by the following equation:


$$\begin{array}{c} f_{\text{2}}=50\times log\left\{{\left[1+\frac{\text{1}}{n}\sum (R_{t}-T_{t}{)}^{\text{2}}\right]}^{-0.5}\times 100\right\} \end{array}$$

where n represents the number of time points, R_t_ is the reference drug release at time point t, and T_t_ is the test drug release at time point t. If the similarity factor (*f*_2_) is greater than or equal to 50, the two drug release profiles are considered similar.

## 3. Results

### 3.1. Determination of theoretical release profile

The biphasic release TIT system should deliver the necessary drug quantity in accordance with predetermined release kinetics, ensuring an effective plasma concentration. To achieve this, the formulation must be designed to release the drug in a controlled, consistent, and reproducible manner. By considering the biopharmaceutic and pharmacokinetic characteristics of the drug, the desired release profile can be established.

The theoretical release of the drug from the TIT system over various time intervals was calculated based on the pharmacokinetic data available for KT. The KT TIT system consists of an outer IR layer and an ER core. In an ideal condition, the ER core will release 0.5 mg (0.94/2 = 0.5) of its total drug content within the first 0.5 h. When combined with the IR layer formulation dose, this should result in a total dose of 10 mg for the first 0.5 h, consistent with the standard dose of a KT tablet. Based on this assumption, the dose of the ER core formulation is calculated to be 22.6 mg (0.94*24 = 22.6), while the dose of the outer IR layer is 9.4 mg (32–22.6 = 9.4).

Consequently, the theoretical release profile of KT from the ER core tablet, expressed as a percentage of the total 22.6 mg dose, is as follows: 2.1% at 0.5 h, 4.2% at 1 h, 8.3% at 2 h, 16.7% at 4 h, 25.0% at 6 h, 33.4% at 8 h, 41.7% at 10 h, 50% at 12 h, 58.4% at 14 h, 66.8% at 16 h, 75.1% at 18 h, and 100% at 24 h. This release profile serves as the reference for comparison with the drug release profiles of the test formulations.

### 3.2. Drug-excipient interaction study

**Fig 2:**
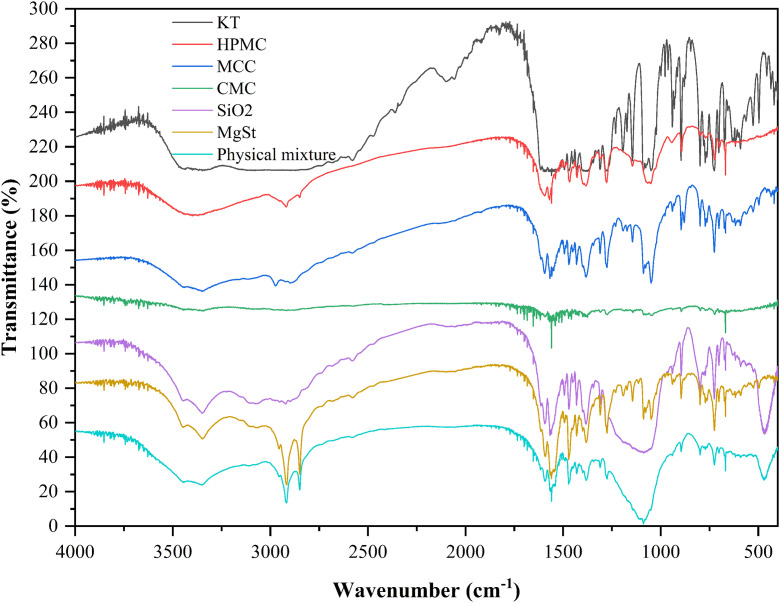
Fourier transform infrared (FTIR) spectrums of ketorolac tromethamine, different excipients and their physical mixture.

**Fig 3:**
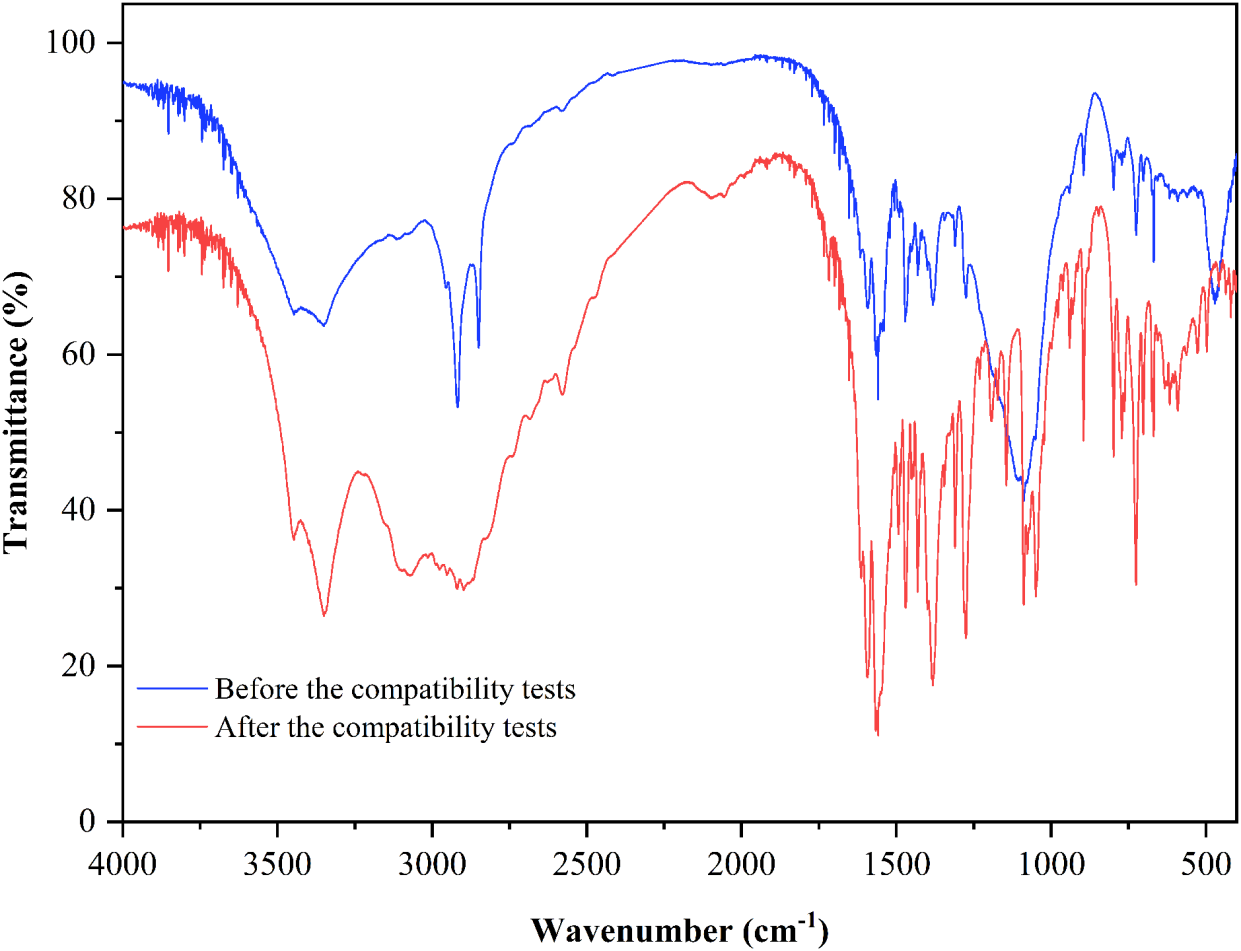
Fourier transform infrared (FTIR) spectrums of physical mixture (ketorolac tromethamine and different excipients) before and after the stressed treatment.

FTIR spectroscopy was employed to investigate potential chemical interactions between ketorolac tromethamine (KT) and selected pharmaceutical excipients. As illustrated in Fig 2, the FTIR spectrum of pure KT exhibits characteristic absorption bands, notably the O-H and N-H stretching vibrations of the related groups appearing broad and strong band around 3500−2800 cm^-1^, and the aromatic C = C stretching near 1600 cm^-1^ (1495, 1469 and 1431 cm^-1^). The spectra of individual excipients, such as the broad O-H stretching bands of HPMC and MCC around 3300−3400 cm^-1^, and the carboxylate bands of CMC near 1600 cm^-1^, align well with their respective chemical structures [23]. Importantly, the spectrum of the physical mixture exhibits a simple superposition of the individual component spectra, without any significant disappearance, shift, or appearance of new peaks. This observation suggests the absence of strong chemical interactions, such as salt formation or covalent bond breaking or formation, under ambient conditions. To further assess compatibility under stressed conditions, the physical mixture was subjected to accelerated stability testing at 40°C and 75% RH for 15 days. Comparative spectra in Fig 3 reveal no notable changes in the characteristic peaks of KT or the excipients post-testing. The stability of the carbonyl and aromatic bands of KT confirms that no detectable chemical degradation or interaction occurred during accelerated stability testing, indicating preliminary chemical compatibility.

**Fig 4:**
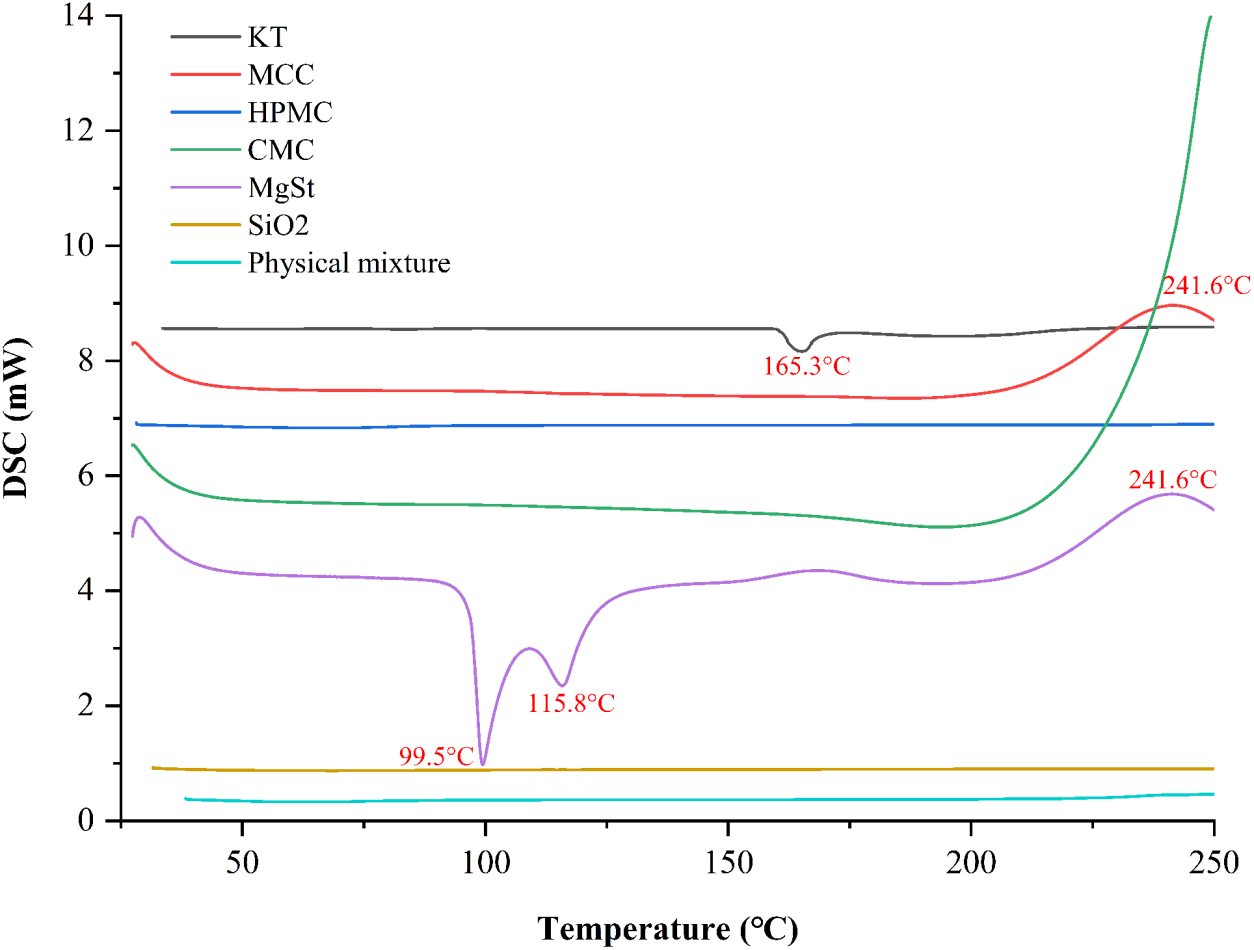
Differential scanning calorimetry (DSC) thermograms of ketorolac tromethamine, different excipients and their physical mixture.

**Fig 5:**
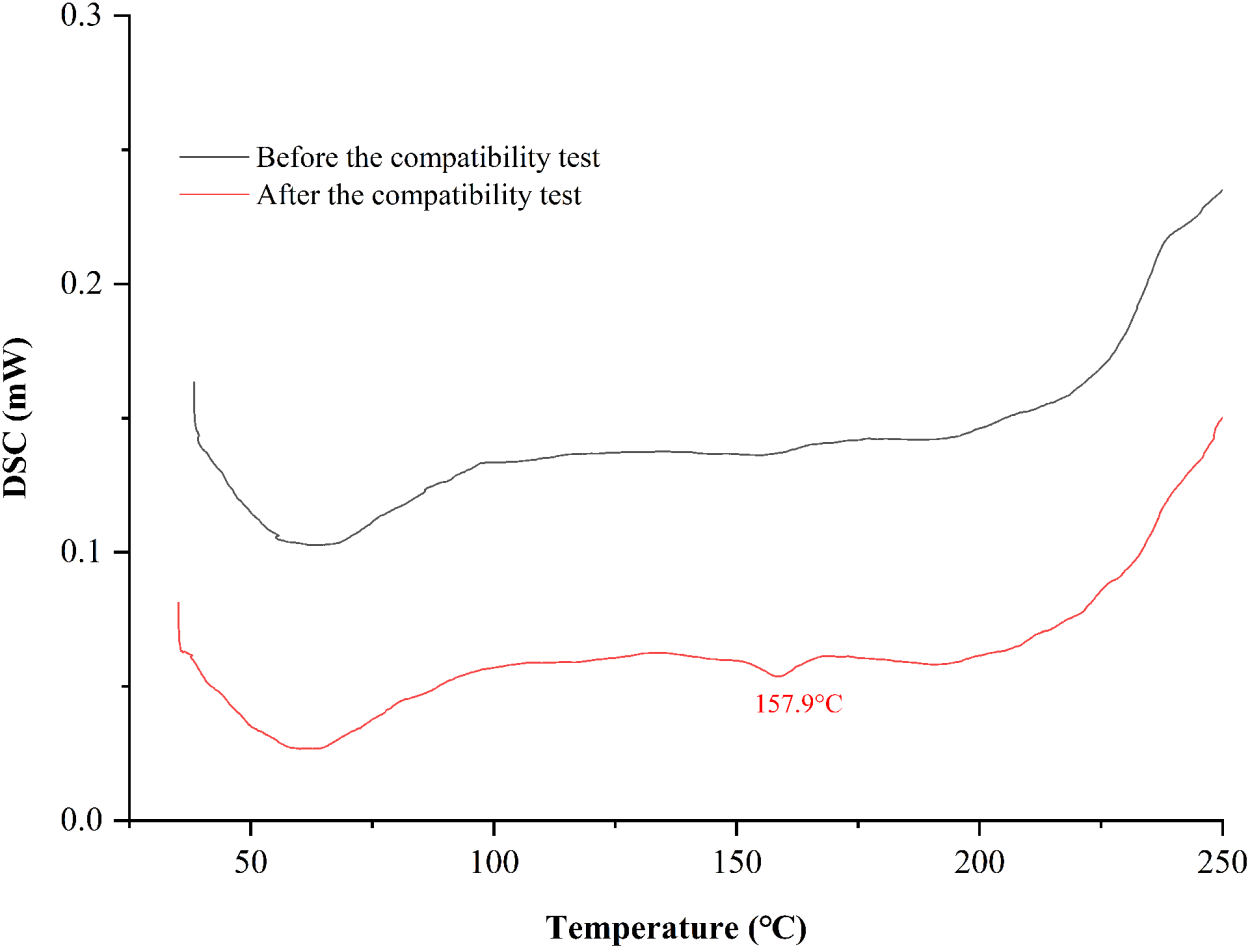
Differential scanning calorimetry (DSC) thermograms of physical mixture (ketorolac tromethamine and different excipients) before and after the stressed treatment.

DSC was utilized to investigate the thermal behavior and potential physical interactions of the components. The thermogram of pure KT, as shown in Fig 4, exhibits a sharp endothermic peak at approximately 165.3°C, corresponding to its melting point and indicative of its crystalline nature. The excipients, including CMC and MCC, demonstrate broad endothermic events around 100°C, which may attributable to water loss [24]. In the physical mixture (Fig 5), the melting endotherm of KT remains detectable (approximately 158°C in the mixture). This phenomenon is likely due to the dilution effect and potential weak physical interactions, such as hydrogen bonding, between KT and the polymeric excipients (e.g., HPMC and MCC), rather than indicating incompatibility. Fig 5 compares the DSC thermograms of the physical mixture before and after the compatibility test. The persistence of the KT melting event after stability testing, without the emergence of new thermal events, is significant and indicates that no substantial solid-state changes, such as polymorphic transformations or amorphous-crystalline phase transitions, were induced by the accelerated conditions. The thermal stability further supports the physical compatibility of the mixture.

**Fig 6:**
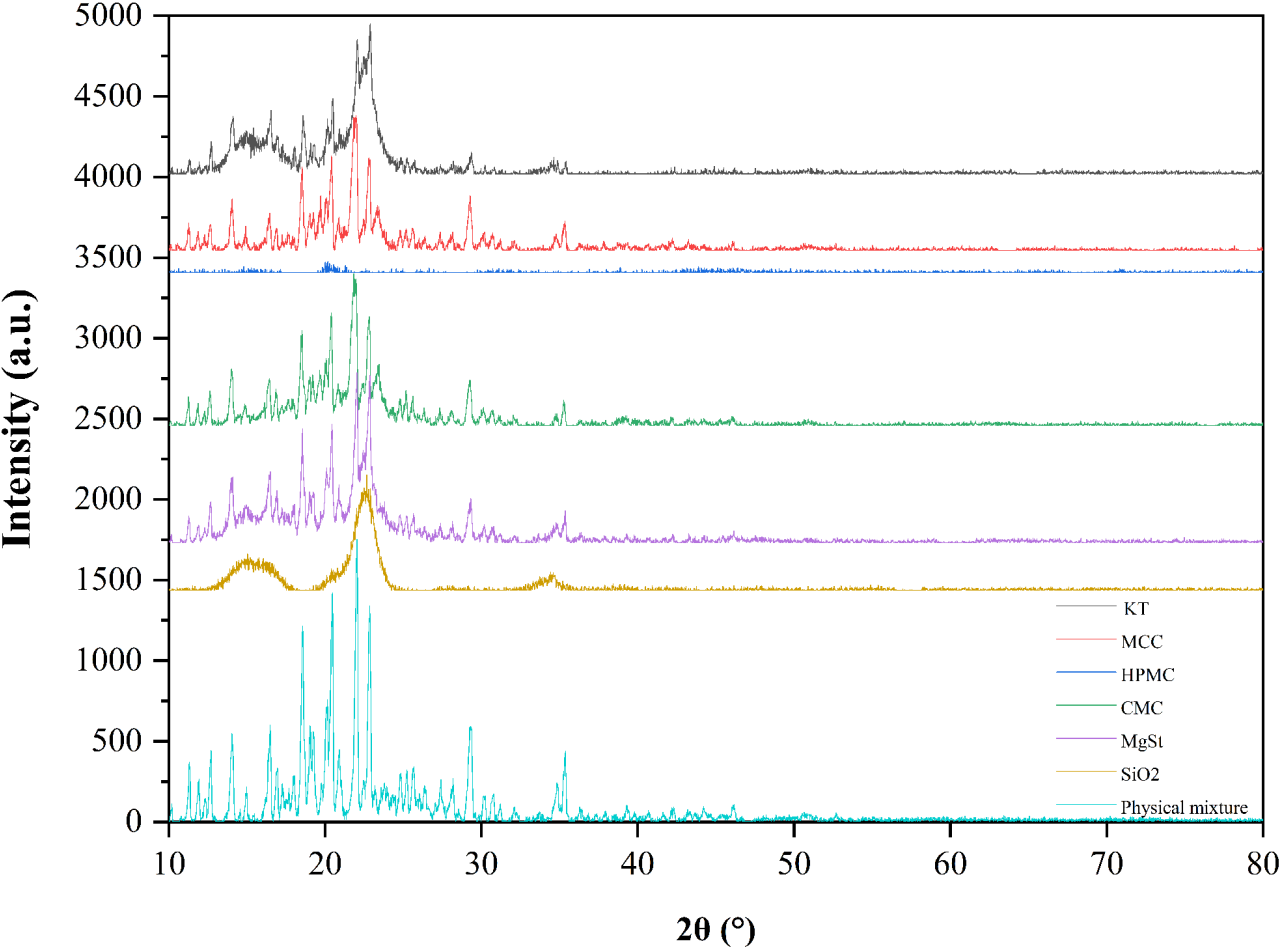
Powder X-ray diffraction (PXRD) diffractograms of ketorolac tromethamine, different excipients and their physical mixture.

**Fig 7:**
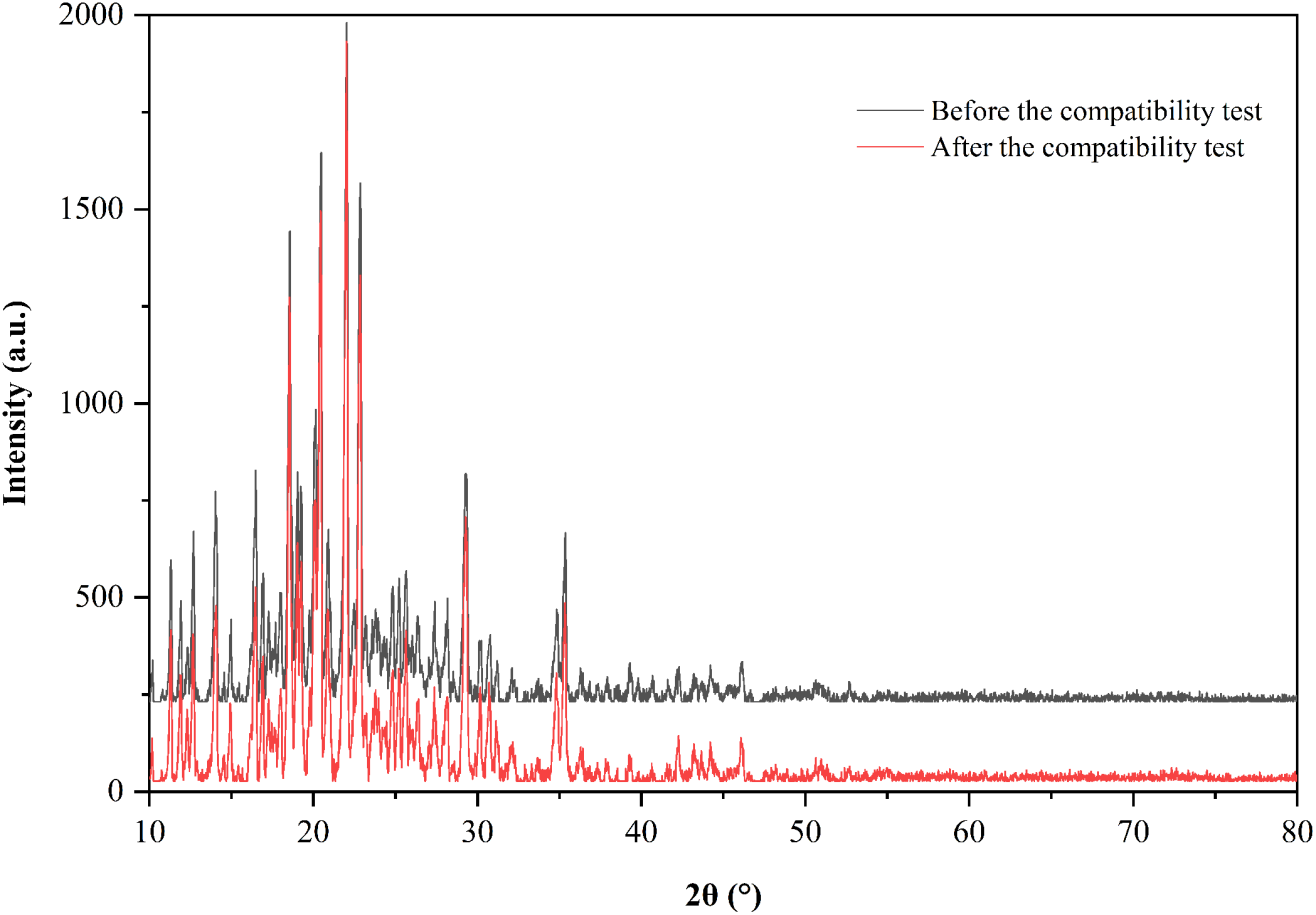
Powder X-ray diffraction (PXRD) diffractograms of physical mixture (ketorolac tromethamine and different excipients) before and after the stressed treatment.

PXRD was employed to monitor changes in the crystalline state of KT within the formulation. The diffractogram of pure KT presented in Fig 6 displays some sharp, distinct peaks, confirming its semi-crystalline nature. Conversely, the diffractograms of excipients such as HPMC and SiO_2_ exhibit a characteristic “halo” pattern typical of amorphous or semi-crystalline materials [25]. The diffractogram of the physical mixture reveals the characteristic peaks of KT, albeit with reduced intensity due to dilution. The preservation of these pivotal diffraction peaks confirms that KT retains its crystalline identity upon simple blending with the excipients. The stability of this crystalline state was further corroborated by the comparative PXRD patterns in Fig 7. The diffractograms of the mixture before and after the 15-day stressed test are virtually identical. The absence of any new peaks or the disappearance of existing KT peaks indicate that no recrystallization, polymorphic conversion, or amorphization occurred during storage. This provides strong evidence for the physical stability and solid-state compatibility of KT with the excipient blend.

By integrating the findings from FTIR, DSC, and PXRD analyses, we achieve a comprehensive assessment of the compatibility between KT and the studied excipients (HPMC, MCC, CMC, SiO_2_, and MgSt). FTIR confirmed the absence of chemical interactions. DSC indicated minor physical interactions but showed no loss of the drug’s fundamental melting behavior or the formation of new phases following stability testing. PXRD unequivocally established that the crystalline integrity of KT was maintained throughout the accelerated stability study.

In conclusion, the integrated data from all three techniques consistently demonstrate that KT is physically and chemically compatible with the selected excipients under the investigated conditions. This compatibility justifies their selection for the subsequent development of a stable solid dosage form.

### 3.3. Evaluation of physical parameters of powders and tablets

The flow properties of granules were assessed using the Carr’s index, Hausner ratio, and angle of repose (Table 5). A Carr’s index value below 10 indicates excellent flow properties, while values greater than 38 suggest poor flow. The Hausner ratio typically ranges from 1.00 to 1.11 for free-flowing materials, with values above 1.60 indicating poor flow properties [26]. In general, an angle of repose below 30° indicates free-flowing material, whereas values exceeding 40° suggest poor flow. The Carr’s index, Hausner ratio, and angle of repose for all powders were found to range from 10.7 to 14.1, 1.12 to 1.16, and 26° to 28°, respectively, indicating that the powders exhibited free-flowing properties and were suitable for tablet compression.

**Table 5:** Physical properties of the powders for extended-release core tablets.

Formulation code	Bulk density (g/cm³)	Tapped density (g/cm³)	Carr’s index	Hausner ratio	Angle of repose (°)
F1	0.4838 ± 0.0046	0.5631 ± 0.0016	14.1 ± 1.8	1.16 ± 0.03	28 ± 4
F2	0.4803 ± 0.0042	0.5567 ± 0.0018	13.7 ± 1.7	1.16 ± 0.03	27 ± 4
F3	0.4654 ± 0.0039	0.5371 ± 0.0013	13.3 ± 1.6	1.15 ± 0.02	27 ± 3
F4	0.4591 ± 0.0037	0.5286 ± 0.0015	13.1 ± 1.7	1.15 ± 0.03	27 ± 3
F5	0.4593 ± 0.0042	0.5187 ± 0.0019	11.5 ± 1.5	1.13 ± 0.04	27 ± 4
F6	0.4752 ± 0.0044	0.5409 ± 0.0014	12.1 ± 1.6	1.14 ± 0.03	27 ± 3
F7	0.4573 ± 0.0041	0.5123 ± 0.0016	10.7 ± 1.3	1.12 ± 0.03	26 ± 3
F8	0.4762 ± 0.0035	0.5339 ± 0.0014	10.8 ± 1.6	1.12 ± 0.02	26 ± 3
F9	0.4476 ± 0.0036	0.4931 ± 0.0017	9.2 ± 1.2	1.10 ± 0.02	26 ± 3

As showed in Table 6, the percentage of weight variation for all prepared tablets was within the acceptable limit of ±5% (w/w), which is considered acceptable for uncoated tablets. The friability test results showed that the weight loss for all formulations was below 1%, indicating that all formulations passed the test. Furthermore, the hardness of all tablets exceeded 44 N, suggesting that the tablets possess sufficient mechanical strength for subsequent pressing. The drug content of all formulations ranged from 96% to 98%, confirming their compliance with the required specification (95%−105%).

**Table 6:** Physical properties of extended-release core tablets of ketorolac tromethamine.

Formulation code	Hardness (N)	Diameter (mm)	Thickness (mm)	Weight (mg)	Friability (%)	Drug Content (%)
F1	50 ± 6	8.0 ± 0.1	2.9 ± 0.2	119 ± 4	0.37 ± 0.03	98.2 ± 1.6
F2	49 ± 5	8.0 ± 0.1	3.0 ± 0.2	121 ± 3	0.31 ± 0.02	97.1 ± 1.7
F3	47 ± 6	8.0 ± 0.1	2.9 ± 0.3	118 ± 3	0.37 ± 0.03	97.2 ± 1.4
F4	48 ± 7	8.0 ± 0.1	3.1 ± 0.3	120 ± 2	0.49 ± 0.04	98.0 ± 2.0
F5	49 ± 5	8.0 ± 0.1	3.1 ± 0.2	119 ± 3	0.45 ± 0.04	97.6 ± 1.5
F6	46 ± 8	8.0 ± 0.1	3.0 ± 0.2	122 ± 3	0.41 ± 0.03	98.4 ± 1.6
F7	47 ± 6	8.0 ± 0.1	3.2 ± 0.3	121 ± 4	0.49 ± 0.04	97.5 ± 1.5
F8	45 ± 6	8.0 ± 0.1	3.1 ± 0.1	122 ± 3	0.41 ± 0.03	98.2 ± 1.8
F9	44 ± 5	8.0 ± 0.1	2.9 ± 0.2	117 ± 4	0.47 ± 0.04	96.3 ± 1.2

### 3.4 In vitro drug release studies of core ER tablet

**Fig 8:**
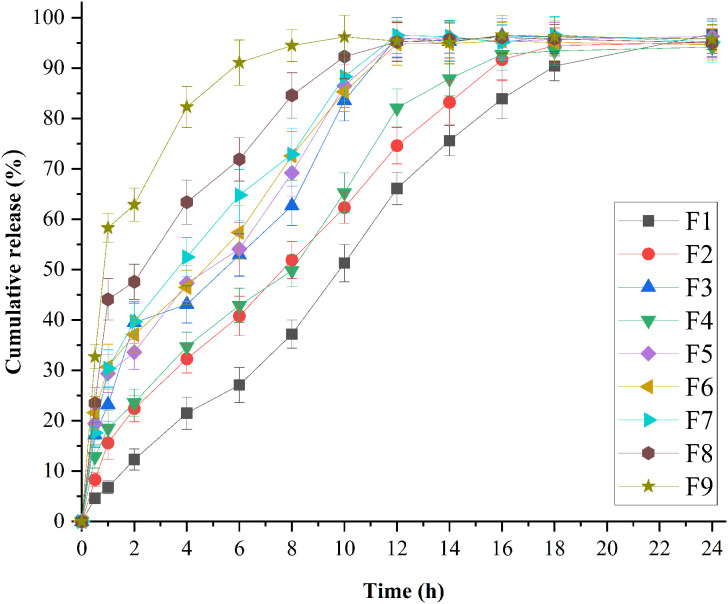
*In vitro* drug release profiles of ketorolac tromethamine from different extended-release core tablets (n = 6).

The drug release from the tablets of all factorial formulations was measured at predetermined time intervals (Fig 8), and the obtained data were compared with the theoretical release profile (used as a reference) to calculate the similarity factor (*f*_2_). As presented in Table 7, the similarity factor analysis revealed that the drug release profile of F1 (*f*_2_ = 51) most closely matched the theoretical release profile. A similarity factor (*f*_2_) greater than 50 indicates that the drug release profile of the test tablets is comparable to that of the reference. Therefore, F1 exhibited the best alignment with the theoretical release pattern among the formulations tested.

**Table 7:** Analysis of *in vitro* release data of extended-release core tablets of ketorolac tromethamine.

Formulation code	*f*_2_ *	Zero order	First order	Higuchi	Korsmeyer–Peppas
F1	51	Y = 3.7407 + 4.5452*X R^2^ = 0.9654	Y = 188.25*(1-exp(−0.034*X)) R^2^ = 0.9820	Y = 22.67*(X^(1/2))-15.01 R^2^ = 0.9402	Y = 7.69*(x^0.831) R^2^ = 0.9749
F2	39	Y = 13.0404 + 4.3565*X R^2^ = 0.9264	Y = 116.97*(1-exp(−0.083*X)) R^2^ = 0.9840	Y = 22.55*(X^(1/2))-7.09 R^2^ = 0.9721	Y = 15.35*(x^0.611) R^2^ = 0.9760
F3	28	Y = 24.7521 + 4.1578*X R^2^ = 0.8219	Y = 102.66*(1-exp(−0.157*X)) R^2^ = 0.9547	Y = 22.52*(X^(1/2))+2.94 R^2^ = 0.9442	Y = 27.10*(x^0.7442) R^2^ = 0.9486
F4	36	Y = 15.6471 + 4.2821*X R^2^ = 0.9029	Y = 111.93*(1-exp(−0.095*X)) R^2^ = 0.9698	Y = 22.33*(X^(1/2))-4.55 R^2^ = 0.9610	Y = 17.59*(x^0.568) R^2^ = 0.9626
F5	27	Y = 26.7385 + 4.0779*X R^2^ = 0.8112	Y = 101.15*(1-exp(−0.145*X)) R^2^ = 0.9654	Y = 22.25*(X^(1/2))+4.94 R^2^ = 0.9453	Y = 29.14*(x^0.419) R^2^ = 0.9533
F6	26	Y = 28.1532 + 3.9490*X R^2^ = 0.8004	Y = 99.067*(1-exp(−0.184*X)) R^2^ = 0.9493	Y = 21.64*(X^(1/2))- + 6.79 R^2^ = 0.9411	Y = 30.61*(x^0.98) R^2^ = 0.9528
F7	25	Y = 28.9669 + 3.9769*X R^2^ = 0.7831	Y = 98.88*(1-exp(−0.199*X)) R^2^ = 0.9578	Y = 21.98*(X^(1/2))+6.98 R^2^ = 0.9362	Y = 31.62*(x^0.392) R^2^ = 0.9515
F8	22	Y = 38.5484 + 3.4985*X R^2^ = 0.6900	Y = 93.95*(1-exp(−0.349*X)) R^2^ = 0.9557	Y = 20.15*(X^(1/2))-17.07 R^2^ = 0.8961	Y = 42.23*(x^0.2954) R^2^ = 0.9585
F9	18	Y = 51.1379 + 2.8778*X R^2^ = 0.5149	Y = 94.39*(1-exp(−0.725*X)) R^2^ = 0.9763	Y = 17.71*(X^(1/2))+30.51 R^2^ = 0.7634	Y = 56.76*(x^0.199) R^2^ = 0.9358

*Compared with theoretical release.

Mathematical models, including zero-order, first-order, Higuchi, and Korsmeyer-Peppas kinetics, were applied to all formulations, as shown in Table 7. Among these models, the *in vitro* drug release of F1 was best described by the first-order model (R^2^ = 0.9820), followed by the Korsmeyer-Peppas model (R^2^ = 0.9749), zero-order model (R^2^ = 0.9654), and Higuchi’s equation (R^2^ = 0.9402).

In the first-order model, drug release from dosage forms containing water-soluble drugs in porous matrices follows a profile where the rate of release per unit time decreases proportionally with the amount of drug remaining in the system. The Korsmeyer-Peppas model is widely used in drug release analysis from polymeric matrices, as it demonstrates a linear relationship between the logarithm of the released drug amount (Log Mt/M0) and the logarithm of time (Log t). Additionally, Table 7 presents the diffusion exponent (n), a key parameter for identifying the transport mechanism within the polymer matrix. A diffusion exponent of 0.45 or lower suggests a Fickian diffusion mechanism (Class I transport), while values between 0.45 and 0.89 typically indicate non-Fickian diffusion, involving both diffusion and erosion in the release process. An n value greater than 0.89 suggests a dominant erosion-driven release (Class II or Super Class II transport) [27,28]. In this case, the diffusion exponent was found to be 0.831, indicating that the release kinetics of the F1 ER core tablet are primarily governed by a combination of diffusion and erosion mechanisms.

### 3.5. Formulation optimization of core ER tablet

The effect of polymer ratios on the drug release performance of ER core-tablets was analyzed using a 3^2^ Full Factorial Design with RSM. Two independent variables, the amounts of HPMC K100M (X_1_) and HPMC K4M (X_2_), were evaluated in a three-level factorial design, resulting in 9 distinct formulations (Table 4). The *in vitro* drug release characteristics were assessed by two dependent variables: Y_1_ (drug release at 0.5 h) and Y_2_ (drug release at 12 h).

ANOVA for the linear model of Y_1_ demonstrated that both HPMC K100M (X_1_) and HPMC K4M (X_2_) significantly influenced the drug release at 0.5 h (Table 8). The Model F-value of 78.31 (p < 0.0001) demonstrates high significance. The individual factors, A (−0.73) and B (−0.61), also had significant effects, with p-values of < 0.0001 and 0.0002, respectively.

**Table 8:** ANOVA for linear model of Y_1_.

Source	Sum of Squares	df	Mean Square	F-value	p-value	
**Model**	539.11	2	269.55	78.31	< 0.0001	significant
A-HPMC K100M	318.28	1	318.28	92.47	< 0.0001	
B-HPMC K4M	220.83	1	220.83	64.16	0.0002	
**Residual**	20.65	6	3.44			
**Cor Total**	559.76	8				

The fitted model equation for Y_1_ is:


$$\begin{array}{c} {\text{Y}}_{\text{1}}\;=\text{ 44}.\text{23 }-\;{\text{0}.\text{73X}}_{\text{1}}-{\text{0}.\text{61X}}_{\text{2}} \end{array}$$

The model’s goodness of fit was evaluated using R^2^, which was 0.9631, indicating that the model explains 96.31% of the variance in the response. The Predicted R^2^ (0.9201) is in reasonable agreement with the Adjusted R^2^ (0.9508), with a difference of around 0.3, confirming that the model has good predictive capability. Additionally, the Adeq Precision value of 24.927 (well above the threshold of 4) indicates an adequate signal, meaning the model can be confidently used to navigate the design space.

For the drug release at 12 h (Y_2_), a two-factor interaction (2FI) model was applied (Table 9), as indicated by the significant interaction term between HPMC K100M (X_1_) and HPMC K4M (X_2_). The Model F-value of 17.67 (p = 0.0043) demonstrates the significance of the model, with all terms (A, B, and AB) having p-values less than 0.05, confirming their importance in determining the drug release at 12 h. The significant interaction term (AB, p = 0.0145) reveals that the combined effect of the two polymers on drug release at 12 h is not simply additive but interactive.

**Table 9:** ANOVA for two-factor interaction (2FI) model of Y_2_.

Source	Sum of Squares	df	Mean Square	F-value	p-value	
**Model**	951.91	3	317.30	17.67	0.0043	significant
A-HPMC K100M	421.68	1	421.68	23.48	0.0047	
B-HPMC K4M	288.43	1	288.43	16.06	0.0102	
AB	241.80	1	241.80	13.46	0.0145	
**Residual**	89.81	5	17.96			
**Cor Total**	1041.72	8				

The fitted model equation for Y_2_ is:


$$\begin{array}{c} {\text{Y}}_{\text{2}}\;=\text{ 88}.\text{03 }+{\;\text{0}.\text{72X}}_{\text{1}}\;+\;{\text{0}.\text{86X}}_{\text{2}}\;-\;{\text{0}.\text{08X}}_{\text{1}}\;{\text{X}}_{\text{2}} \end{array}$$

The R^2^ for this model was 0.9138, which suggests that 91.38% of the variation in the response is explained by the model. The Adjusted R^2^ of 0.8621 and the Predicted R^2^ of 0.8332 showing reasonable agreement with a difference between them being less than 0.3. The Adeq Precision value of 11.438 (well above the threshold of 4) indicates an adequate signal for navigating the design space.

**Fig 9:**
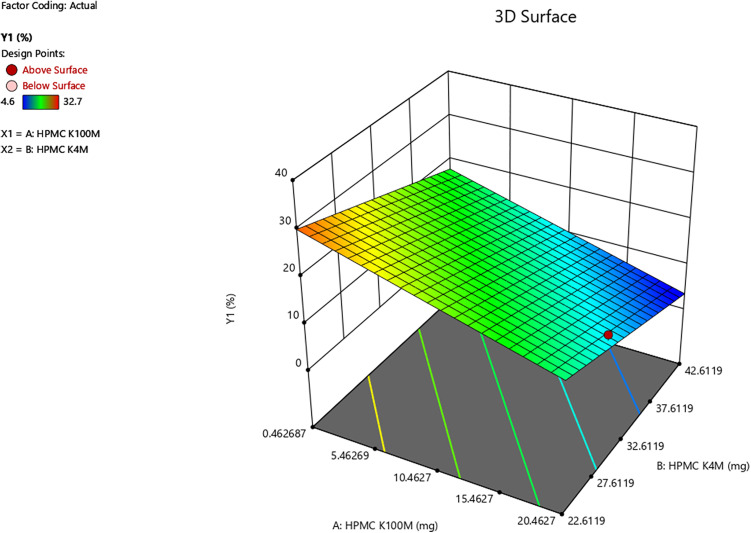
3D surface plot illustrating drug release at 0.5 h (Y_1_).

**Fig 10:**
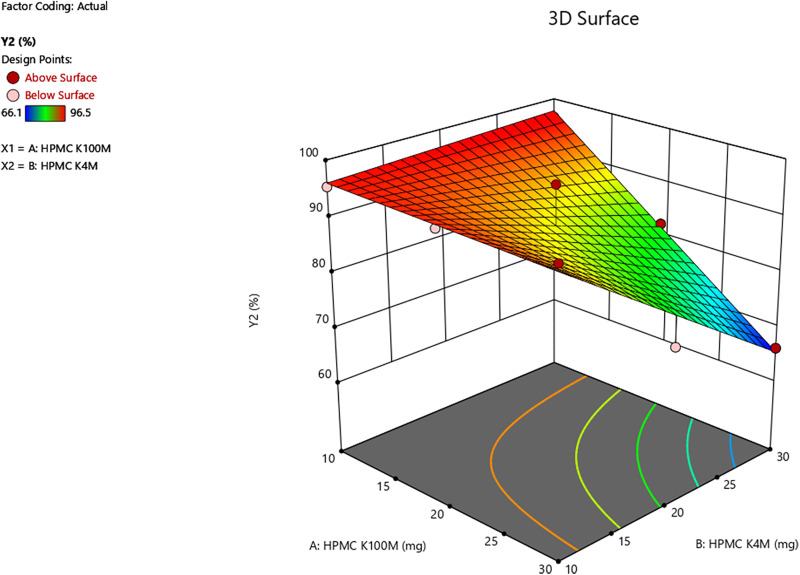
3D surface plot illustrating drug release at 12 h (Y_2_).

The 3D graphical plots of the regression equations for Y_1_ (Fig 9) and Y_2_ (Fig 10), obtained via Design-Expert software, reveal a similar pattern. The 3D surface plot for Y1 shows a gradual decrease in drug release at 0.5 h as both HPMC K100M and HPMC K4M concentrations increase, consistent with the formation of a more viscous and robust gel layer. The surface for Y2 exhibits a slight curvature, reflecting the significant interaction between the two polymers in sustaining drug release over 12 h.

The optimization criteria, aiming to minimize Y_1_ and maintain Y_2_ within the range of 50–75%, were utilized to refine the polymer ratios and achieve the most suitable release characteristics. The desired responses, Y_1_ (4.5%) and Y_2_ (66.1%), were predicted using the point prediction method in Design-Expert software, with X1 set at 29.7 mg and X_2_ at 29.9 mg. Upon rounding the recommended values for X_1_ and X_2_, the optimal formulation, F1, was prepared at the ‘+1’ (30 mg) level for both factors, X_1_ (HPMC K100M content) and X_2_ (HPMC K4M content). This formulation was identified as the optimum for ER core tablet.

Although several formulations showed low Y_1_ values (e.g., F1: 4.6%, F2: 8.3%), the optimization criteria required minimizing Y_1_ while keeping Y_2_ within 50–75%. F1 met both targets with Y_1_ = 4.6% and Y_2_ = 66.1%, closely matching the predicted values (4.5% and 66.1%) from the response surface model. In contrast, F2 had Y_2_ = 74.6% (still within range) but higher Y_1_ (8.3%), and F3-F9 exhibited Y_2_ > 75% or Y_1_ > 10%. Therefore, F1 was selected as the optimal formulation.

To validate the reproducibility of the optimized formulation (F1), three independent batches were prepared under identical conditions. *In vitro* drug release profiles demonstrated mean cumulative releases of 4.7 ± 1.8% at 0.5 h, 65.1 ± 3.4% at 12 h, and 96.6 ± 2.9% at 24 h (mean ± SD). The observed release data showed close agreement with model predictions, confirming the reliability of the RSM for formulation optimization in this study.

### 3.6. Characterization of tablet-in-tablets

In this study, we developed and characterized TIT formulations of KT based on an ER core tablet (F1). The corresponding TIT formulations (F10, F11, and F12) were produced with outer shell weights of 300 mg, 331 mg, and 362 mg, respectively, using a 12 mm punch. These formulations were characterized for thickness, hardness, mass, friability, disintegration time, and drug content (Table 10).

**Table 10:** Results of quality characteristics of tablet-in-tablet.

Formulation code	Thickness (mm)	Hardness (N)	Mass (mg)	Friability (%)	Disintegration Time (s) (Outer layer)	Drug Content (%)
F10	4.7 ± 0.1	56 ± 7	421 ± 5	3.74 ± 0.58	42 ± 4	101.5 ± 1.1
F11	5.2 ± 0.1	75 ± 6	450 ± 5	0.59 ± 0.18	48 ± 4	97.8 ± 1.3
F12	5.7 ± 0.1	79 ± 6	481 ± 6	0.53 ± 0.16	50 ± 5	99.6 ± 1.2

The outer shell weight significantly influenced the structural integrity of the TIT tablets. Hardness increased with outer shell weight, with F10 showing the lowest hardness (56 N), followed by F11 (75 N) and F12 (79 N). Batch F10, with the lightest outer shell, exhibited higher friability (3.74 ± 0.58%), exceeding the acceptable limit of 1%, and occasional splitting, likely due to insufficient bonding between the core and outer shell. In contrast, F11 and F12 showed improved hardness (75–80 N) and significantly lower friability (0.59 ± 0.18% and 0.53 ± 0.16%), indicating better encapsulation and structural integrity. These findings suggest that a balance between excipient use and tablet strength is crucial for ensuring durability and effective drug release.

The disintegration times of all TIT formulations were within acceptable limits, with F10 disintegrating in 42 s, F11 in 48 s, and F12 in 50 s. The shorter disintegration time for F10 correlates with its lower hardness and higher friability, while the longer times for F11 and F12 reflect the impact of increased outer shell weight.

**Fig 11:**
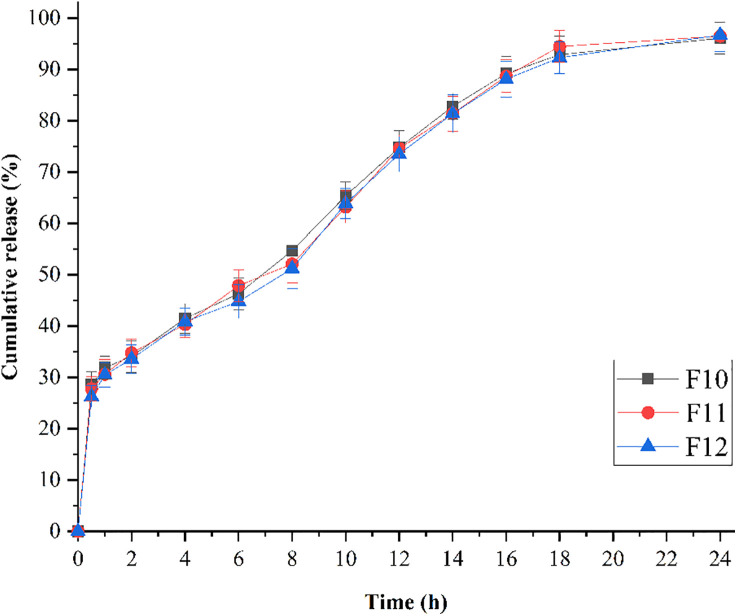
*In vitro* drug release profiles of table-in-tablets with different out shell formulations (F10-F12).

The rapid disintegration of the outer layer (within 50 s for all formulations) confirms its immediate-release function, which is critical for achieving the initial burst release observed in dissolution profiles (Fig 11). The disintegration time correlates inversely with the hardness and outer layer weight: F10 (lightest shell) disintegrated fastest (42 s) but exhibited high friability, while F11 and F12 showed slightly longer disintegration times (48–50 s) with improved mechanical integrity.

The drug release profiles for all three formulations were similar (*f*_2_ values between any two formulations were greater than 50), exhibiting an initial burst release of approximately 30%, followed by sustained release over 18 h (Fig 11). Importantly, the release profiles of the TIT formulations did not differ significantly from that of the ER core tablet (F1), indicating that the outer layer did not substantially influence the core’s extended-release mechanism. The outer layer primarily facilitated an initial controlled release without altering the release characteristics of the core tablet.

The outer layer contained CMC as a disintegrant, which facilitated rapid disintegration through swelling, erosion, and pore formation [29]. The addition of MCC enhanced disintegration by promoting fluid ingress [30]. A 3.0% CMC concentration in the outer layer facilitated fast disintegration, with F10 having the shortest disintegration time. F11 and F12, with larger outer shells, had slightly longer disintegration times.

Upon immersion in the dissolution medium, both layers of the TIT system begin to release drug simultaneously. The outer IR layer disintegrates rapidly (within 50 s), releasing its immediate dose (~30% of total) within the first 30 min. Concurrently, the ER core matrix hydrates and forms a gel layer, initiating a slow, sustained release that continues over 24 h. This biphasic release behavior ensures an initial therapeutic concentration followed by prolonged maintenance.

**Fig 12:**
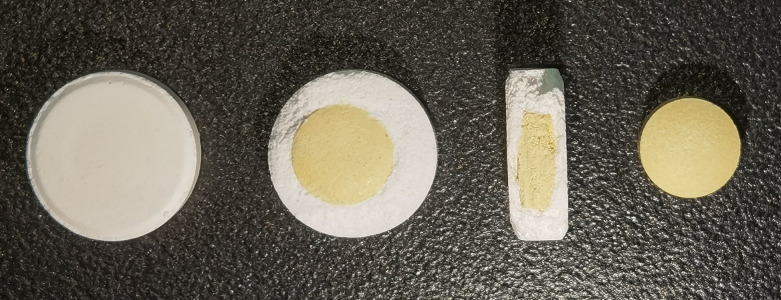
Appearances of F11 table-in-tablets. Yellow pigments were added in the core-tablet in this picture.

Among the formulations, F11, with the 331 mg outer shell (Fig 12), was the optimal choice, offering a good balance of tablet hardness, friability, and disintegration time, while maintaining efficient drug release. Compared to F12, F11 required fewer excipients, making it a more economical option without sacrificing performance. F11’s enhanced structural integrity and consistent release profile suggest it is a promising candidate for further development in KT TIT systems.

This study highlights the importance of carefully balancing outer shell weight, hardness, and excipient composition in the design of tablet-in-tablet (TIT) formulations, ultimately achieving a system that delivers the intended biphasic release profile. Although F10 exhibited certain mechanical limitations, both F11 and F12 provided robust alternatives, with F11 emerging as the optimal formulation due to its efficient excipient utilization, excellent disintegration properties, and strong mechanical integrity. The optimized core (F1) demonstrated excellent agreement with the theoretical release profile (*f*_2_ = 51), validating the formulation strategy, and incorporation into the final TIT (F11) successfully combined rapid initial release with sustained drug delivery over 24 h. Importantly, the presence of the outer layer did not significantly alter the drug release kinetics of the extended-release core, thereby preserving its therapeutic advantages while meeting predefined pharmacokinetic objectives. Future studies should focus on further excipient optimization and *in vivo* evaluation to confirm the clinical performance and efficacy of the developed TIT system.

## 4. Discussion

### 4.1. Formulation design

The successful development of a robust biphasic-release TIT system hinges on the rational design of the ER core, ensuring its stability, manufacturability, and desired drug release profile. In this study, the theoretical release profile for the KT ER core was first established based on pharmacokinetic parameters, targeting a specific zero-order release rate to maintain steady-state plasma concentrations. This profile served as a critical benchmark for formulation optimization. A systematic pre-formulation strategy was employed to guarantee the feasibility of this design. Comprehensive drug-excipient compatibility studies using FTIR, DSC, and PXRD confirmed the absence of detrimental chemical interactions and the maintenance of KT’s solid-state properties within the excipient matrix after stress testing. This foundational compatibility is paramount for ensuring the chemical stability and predictable performance of the final dosage form. It is important to acknowledge the limitations of the 15-day accelerated compatibility study. While this duration is consistent with common practice for preliminary compatibility screening and provides initial evidence of drug-excipient compatibility, it cannot predict degradation pathways that might emerge over extended storage periods. Therefore, the present results should be interpreted as a preliminary screening rather than a definitive stability assessment. Future research should focus on comprehensive stability studies, including long-term (25°C/60% RH for up to 24 months), intermediate (30°C/65% RH for 12 months), and accelerated (40°C/75% RH for 6 months) storage conditions. These studies should evaluate critical quality attributes including drug content, dissolution profile, hardness, friability, and degradation product formation. Additionally, photostability studies and container-closure system compatibility testing would further support the commercial viability of this formulation.

Concurrently, the powder blends for all experimental formulations demonstrated excellent flow properties, which is essential for achieving uniform die filling and consistent tablet weight during compression. The resulting core tablets exhibited satisfactory mechanical strength, low friability, and uniform drug content, meeting pharmacopeial standards and confirming the suitability of the direct compression process for this formulation.

The combination of HPMC K100M (high viscosity) and HPMC K4M (low viscosity) was chosen to achieve a fine-tuned release profile. Both polymers are hydrophilic and form gel matrices, but their different viscosities allow modulation of the gel layer strength and erosion rate. Using two grades of the same polymer minimizes the risk of incompatibility and simplifies regulatory aspects, while still providing a wide range of release kinetics. Preliminary trials confirmed that the mixture could produce release profiles spanning the desired range, whereas single grades alone either released too fast (K4M) or too slow (K100M) to meet the target.

The core of the development was the optimization of the ER matrix using a 3^2^ full factorial design. The results unequivocally demonstrated that the concentrations of HPMC K100M (X_1_) and HPMC K4M (X_2_) were critical factors controlling the drug release. The range for HPMC K100M and HPMC K4M (10–30 mg) was selected based on preliminary single-factor experiments (S1 Appendix) and literature review. In the preliminary studies, formulations containing 5 mg of each polymer exhibited rapid drug release (>80% at 6 h) due to insufficient gel formation, while formulations with 40 mg of each polymer showed incomplete release (<65% at 24 h) and poor tablet hardness. Based on these results, the 10–30 mg range was chosen as it covered formulations with potentially optimal release characteristics. The combination of HPMC K100M (high viscosity, 100,000 cP for 2% solution) and HPMC K4M (lower viscosity, 4,000 cP for 2% solution) was selected to achieve a fine-tuned release profile, as their different viscosities allow modulation of the gel layer strength and erosion rate. Using two grades of the same polymer minimizes the risk of incompatibility and simplifies regulatory aspects, while still providing a wide range of release kinetics.

The statistical models revealed that higher polymer levels significantly retarded the initial drug release (Y_1_), attributable to the formation of a more viscous and robust gel layer that impeded drug diffusion and water penetration. The interaction effect on the 12-hour release (Y_2_) further highlighted the complex, synergistic role of the polymer blend in sustaining drug release over the entire duration. The optimal formulation, F1, was identified and validated. Its release profile (*f*_2_ = 51) closely matched the theoretical target. Kinetic analysis indicated that the drug release from F1 followed first-order kinetics and was best explained by the Korsmeyer-Peppas model (n = 0.831), suggesting an anomalous transport mechanism where drug release is governed by a combination of diffusion through the hydrated polymer matrix and polymer chain relaxation/erosion.

The objective of an ideal ER system here is to achieve zero-order release kinetics. However, the release profile of the optimized formulation (F1) was most accurately modeled by first-order kinetics. This phenomenon is attributed to the inherent release mechanism of the hydrophilic matrix tablet employed in this study. In such matrices, the release rate is concentration-dependent and decreases as the drug diffuses out, which is a recognized limitation of this technology. In contrast, osmotic pump systems are specifically designed to provide a more constant, zero-order release rate [14]. Nevertheless, it is important to recognize that the R^2^ values for the zero-order and first-order fits for F1 are actually quite close (0.9654 vs. 0.9820, respectively, as shown in Table 7), indicating that the optimized formulation also approximates zero-order release reasonably well. The first-order model provides a slightly better statistical description, but the difference is not substantial. The zero-order model was employed as a pharmacokinetic design target to estimate the required maintenance dose and release rate, not as a prediction of the actual release mechanism. The similarity factor (*f*_2_ = 51) between the experimental and theoretical profiles further confirms that the observed release behavior closely matches the predefined pharmacokinetic target, underscoring the practical relevance of this formulation.

### 4.2. Formulation optimization of core ER tablet

The results of this study highlight the significant influence of HPMC K100M and HPMC K4M on the drug release characteristics of ER core-tablets. Both the linear and 2FI models were effective in capturing the effects of these polymers on the drug release at early and late time points (0.5 h and 12 h).

The significant negative coefficients for both HPMC K100M and HPMC K4M in the equation for Y_1_ suggest that higher concentrations of these polymers result in a decrease in drug release at the initial time point (0.5 h). This can be attributed to the increased viscosity and gel formation at higher polymer concentrations, which can retard the initial drug diffusion. The presence of both HPMC K100M and HPMC K4M may create a more cohesive gel matrix, slowing down the release of the drug.

For drug release at 12 h, the 2FI model demonstrates a more intricate interaction between the two polymers. The significant interaction term (X_1_X_2_) indicates that the combined effect of the polymers on drug release is not simply additive, but rather interactive. In this experimental design, the interaction between the two polymers was more pronounced than the effect of each individual factor on Y_2_.

The theoretical release profile specifies a target of 50% at 12 hours. However, the optimization criterion for Y_2_ was empirically set to a range of 50–75%. The rationale for this enhanced target is the typical slowdown of drug release in the latter phase of extended-release matrix systems. Setting a higher, more flexible target for Y_2_ helps ensure that the formulation achieves a near-complete release profile *in vitro*.

The optimized formulation should balance the amount of HPMC K100M and HPMC K4M to achieve the desired release profile. Specifically, a higher concentration of both polymers may be suitable for maintaining a sustained drug release over a longer duration, while lower concentrations may allow for a quicker release at earlier time points.

The core tablet diameter (8 mm) was chosen to accommodate the desired core weight (120 mg) and to allow sufficient space for the outer layer to fully encapsulate it when using a 12 mm final punch. The 12 mm diameter provides an outer layer thickness of approximately 1.5–2 mm (calculated as half the difference between final and core diameters), which was found sufficient for rapid disintegration and mechanical protection in preliminary trials.

Regarding reproducibility and scale-up, the TIT system requires a multilayer tablet press with precise alignment and compression force control. In our study, all batches showed low variability in weight, hardness, and dissolution (RSD < 5%), indicating good laboratory-scale reproducibility. For industrial scale-up, automated feeding systems and real‑time monitoring of core placement can minimize variation. Although the process is more complex than conventional bilayer tableting, it offers advantages in complete core encapsulation and dose flexibility. Similar compression‑coating processes have been successfully scaled up for commercial products, suggesting that with appropriate equipment, the TIT system can be manufactured reproducibly.

### 4.3. Characterization of tablet-in-tablets

The development of TIT formulations represents an innovative approach to optimizing drug delivery, particularly for ER systems. In this study, we successfully characterized TIT formulations of KT by evaluating their physicochemical properties and drug release kinetics. The findings underscore the critical role of outer shell composition in determining tablet integrity, disintegration behavior, and drug release performance.

Our results demonstrate that increasing the outer shell weight significantly enhances tablet hardness while reducing friability. Formulation F10, with the lightest outer shell (300 mg), exhibited insufficient mechanical strength, leading to high friability (3.74%) and occasional splitting—likely due to weak bonding between the core and outer layer. This observation aligns with previous studies showing that insufficient compression force or excipient ratio can compromise tablet integrity [31]. In contrast, formulations F11 (331 mg) and F12 (362 mg) displayed superior hardness (75–79 N) and minimal friability (<1%), ensuring structural integrity during handling and storage. This suggests that an optimal outer shell weight is essential to prevent premature tablet failure while maintaining efficient drug release, consistent with findings on bilayer tablet formulations [20].

The disintegration times of all TIT formulations (42–50 s) remained within acceptable limits, with F10 disintegrating slightly faster due to its lower hardness. Notably, despite differences in outer shell weight, the drug release profiles of all TIT formulations closely mirrored that of the ER core tablet (F1), with an initial burst release (~30%) followed by sustained release over 18 h. The similarity factor (*f*_2_ > 50) confirmed that the outer layer did not interfere with the core’s ER mechanism, functioning primarily as a facilitator for initial controlled release. This is attributed to the inclusion of CMC and MCC in the outer layer, which promote rapid disintegration through swelling and fluid ingress, as previously reported in studies on disintegrant mechanisms [32].

Among the tested formulations, F11 (331 mg outer shell) emerged as the most balanced candidate, offering adequate hardness, low friability, and efficient disintegration without excessive excipient use. While F12 exhibited marginally better mechanical properties, the additional excipients did not significantly improve performance, making F11 a more cost-effective option. The consistent drug release profile of F11 further supports its suitability for clinical development, similar to findings in other studies evaluating optimized TIT systems [20].

The TITs exhibited a controlled and sustained *in vitro* release profile. Approximately 30% of the loaded KT was released within the initial 10 minutes, representing the IR release phase. This was followed by a gradual and consistent release of the remaining drug over 18 h, corresponding to the ER phase. By the end of the 18-hour dissolution study, the cumulative drug release from the TITs exceeded 90%, indicating an ER characteristic that may offer therapeutic benefits through the maintenance of drug concentrations over an extended duration.

It is important to recognize that the present work represents a preclinical formulation development stage. While the *in vitro* release profiles indicate promising potential for sustained drug delivery, the ultimate clinical performance must be confirmed through appropriate *in vivo* pharmacokinetic studies. Such studies are planned as a logical continuation of this research and will be reported separately. These future investigations will evaluate the systemic exposure, bioavailability, and fluctuation index of the TIT system, thereby providing essential data to validate the therapeutic advantages predicted by the *in vitro* release behavior.

This study highlights the importance of optimizing outer shell composition to achieve robust TIT formulations while preserving the therapeutic benefits of the ER core. Future research should focus on refining excipient ratios to enhance tablet durability and investigating *in vivo* performance to validate the pharmacokinetic advantages observed *in vitro*. Additionally, scaling up production and assessing long-term stability will be crucial for translating these findings into commercially viable products.

## 5. Conclusions

A novel biphasic-release TIT system for KT was successfully formulated and developed. The ER core was effectively optimized using a 3^2^ factorial design, identifying the optimal concentrations of HPMC K100M and HPMC K4M to achieve a desired drug release profile. Drug-excipient compatibility screening provided preliminary evidence of formulation stability under accelerated conditions. The final TIT system demonstrated excellent tablet properties, with a rapid-disintegrating outer layer providing an immediate dose and a robust ER core ensuring sustained drug release over 18 hours. The *in vitro* release profile was well-characterized and shown to be consistent and reproducible. This developed KT TIT formulation presents a promising strategy for enhancing therapy by maintaining effective drug concentrations over an extended period, potentially enabling once-daily dosing and improving patient adherence in the management of pain.

## Supporting information

S1 Appendix Experiment data. (XLSX)
